# Associations of criminal justice and substance use treatment involvement with HIV/HCV testing and the HIV treatment cascade among people who use drugs in Oakland, California

**DOI:** 10.1186/s13722-017-0078-9

**Published:** 2017-06-14

**Authors:** Barrot H. Lambdin, Alex H. Kral, Megan Comfort, Andrea M. Lopez, Jennifer Lorvick

**Affiliations:** 1 RTI International, 351 California St, Suite 500, San Francisco, CA 94104 USA; 2 University of California, San Francisco, San Francisco, CA USA; 3 University of Washington, Seattle, WA USA

**Keywords:** Criminal justice, Substance use treatment, HIV, Hepatitis C, People who use drugs, Implementation science

## Abstract

**Background:**

People who smoke crack cocaine and people who inject drugs are at-risk for criminal justice involvement as well as HIV and HCV infection. Compared to criminal justice involvement, substance use treatment (SUT) can be cost-effective in reducing drug use and its associated health and social costs. We conducted a cross-sectional study of people who smoke crack cocaine and people who inject drugs to examine the association between incarceration, community supervision and substance use treatment with HIV/HCV testing, components of the HIV treatment cascade, social and physical vulnerability and risk behavior.

**Methods:**

Targeted sampling methods were used to recruit people who smoke crack cocaine and people who inject drugs (N = 2072) in Oakland, California from 2011 to 2013. Poisson regression models were used to estimate adjusted prevalence ratios between study exposures and outcomes.

**Results:**

The overall HIV prevalence was 3.3% (95% CI 2.6–4.1). People previously experiencing incarceration were 21% (p < 0.001) and 32% (p = 0.001), respectively, more likely to report HIV and HCV testing; and were not more likely to report receiving HIV care or initiating ART. People previously experiencing community supervision were 17% (p = 0.001) and 15% (p = 0.009), respectively, more likely to report HIV and HCV testing; and were not more likely to report receiving HIV care or initiating ART. People with a history of SUT were 15% (p < 0.001) and 23% (p < 0.001), respectively, more likely to report receiving HIV and HCV testing, 67% (p = 0.016) more likely to report HIV care, and 92% (p = 0.012) more likely to report HIV treatment initiation. People previously experiencing incarceration or community supervision were also more likely to report homelessness, trouble meeting basic needs and risk behavior.

**Conclusions:**

People with a history of substance use treatment reported higher levels of HCV and HIV testing and greater access to HIV care and treatment among HIV-positive individuals. People with a history of incarceration or community supervision reported higher levels of HCV and HIV testing, but not greater access to HIV care or treatment among HIV-positive individuals., Substance use treatment programs that are integrated with other services for HIV and HCV will be critical to simultaneously address the underlying reasons drug-involved people engage in drug-related offenses and improve access to essential medical services.

## Background

Since the 1980s, the “War on Drugs” has contributed to drastic increases in incarceration in the United States, including a threefold increase in drug-related arrests and an eightfold increase in the prison population [1–3]. Consequently, the US has the highest documented incarceration rate in the world at 716 inmates per 100,000 residents [4], and an astonishing 4,751,400 people on probation or parole [5]. Because law enforcement policies target people who use drugs, 64.5% of people who are incarcerated experience a substance use disorder; however, only 11% receive any type of substance use treatment while incarcerated [6].

People who smoke crack cocaine (PWSC) and people who inject drugs (PWID) are not only at high risk for incarceration, but they are also at high risk for HIV and hepatitis C virus (HCV) infection. Thus, the estimated HIV prevalence is nearly three times higher among incarcerated populations than the general population (1.5 vs. 0.6%) and HCV prevalence is 9–27 times higher (12–35% compared to 1.3%) [7–9].

Access to care and adherence to treatment are key to reducing morbidity and mortality from HIV and HCV. For HIV, antiretroviral therapy (ART) is associated with improved clinical outcomes, longer survival and secondary prevention of infection, including reduced HIV transmission risk at the community level [10–12]. For HCV, the advent of direct acting antiretroviral (DAA) medications, with a relatively short course of treatment and minimal side effects, has led to cure rates of 90%, and the possibility of virtually eliminating HCV transmission. Benefitting from these therapies, however, requires participation in a series of sequential steps, often referred to as the HIV or HCV treatment cascade [13, 14]. These include diagnosis of HIV/HCV, linkage to care, clinical evaluation, treatment initiation, retention in care, and treatment adherence, with the ultimate goal of making viral load undetectable [13–15].

The lives of people using illicit drugs can be chaotic due to stigma, severe poverty, probation/parole requirements, comorbidities, serious mental illness, and the psychological and clinical effects of the substances they ingest, making it difficult to access and adhere to treatment for HIV or HCV in community settings. Incarceration can provide a point of access for HIV/HCV services [16–20], but this does not always translate to successful navigation of the care continuum [21–25]. Community supervision can provide another opportunity to facilitate access to HIV/HCV services, but this opportunity is often not realized [26, 27].

Research has suggested that substance use treatment (SUT) can effectively address the underlying reasons why many people who use drugs become engaged in drug-related offenses [28]. Further evidence suggests that SUT is cost-effective in reducing drug use and its associated health and social costs, as compared to incarceration [29]. SUT has been shown to help people lower their risk of HIV acquisition and transmission, improve their access and adherence to HIV treatment and reduce their viral load [30–33].

In this cross-sectional study of PWSC and PWID, we examine the association of a history of incarceration, community supervision and substance use treatment with access to HIV/HCV testing, components of the HIV treatment cascade, social and physical vulnerability and risk behavior. We additionally assess for predictors of HIV and HCV status.

## Methods

### Study setting

Our study includes a community-based sample of PWSC and PWID in Oakland, California. Oakland is a racially diverse, mid-sized city in Alameda County with a population of 400,000 people. Alameda County was the first county in the United States to declare a state of emergency in 1998 due to a disproportionally high HIV prevalence among the African American population, an emergency that continues to this day [34]. In 2013, the HIV prevalence was 113 per 100,000 people with 80 new diagnoses per 100,000 among African American men [35, 36]. In addition, the adult incarceration prevalence in Alameda County was 1471 per 100,000 men (nearly double the national average) and 86 per 100,000 women in 2010, and the prevalence of community supervision (probation or parole) was 1580 per 100,000 people, with most people clustered in 6 contiguous zip codes of Oakland [37, 38]. Residents in these areas also carry a disproportionate burden of poor health, low income and unstable housing [39].

### Study population

Using targeted sampling methods [40, 41], an outreach worker recruited participants from July 2011 to July 2013 in street settings within the cluster of zip codes having high community supervision levels [42], and collected data at three easily accessible field sites. From July 2011 to July 2013, an outreach worker recruited a total of 2323 participants in neighborhoods surrounding the three field sites. Inclusion criteria for the study included crack cocaine or injection drug use in the 6 months prior to interview and age ≥18. Drug use was verified by use of a screening instrument that obscured eligibility requirements. Approximately 10% of recruited participants did not meet eligibility criteria, leading to 2072 participants for this study.

Participants engaged in an informed consent process, a quantitative interview and HIV testing, as well as pre- and post-test counseling. The quantitative interview was conducted face-to-face, with interviewers posing items verbally and recording responses in a computer-based personal interviewing system (Blaise^®^, Westat). Rapid testing for HIV infection was conducted using the OraQuick ADVANCE^®^ rapid HIV antibody test. Reactive results on the OraQuick test were confirmed with a second point-of-care test, the Clearview STAT-PAK^®^. Interview staff were trained in HIV testing and counseling as well as data collection techniques. Participants who were HIV antibody positive were eligible for a separate intervention study, complete with a new informed consent process [43].

All study procedures were reviewed and approved by a federally accredited Institutional Review Board at RTI International. Participants received $20 remuneration for their contribution to the research, as well as referrals to medical and social services as appropriate.

### Measures

Outcome variables included measures for HIV/HCV testing, the HIV treatment cascade, social and physical vulnerability, risky injection or sexual behavior, and HIV/HCV status. HCV testing was defined as having ever had an HCV antibody test. Variables for steps of HIV treatment cascade included HIV testing, defined as receiving an HIV antibody testing ever; received HIV care, defined as ever receiving HIV care among those who are HIV positive; and initiated ART, defined as ever starting ART among those who are HIV positive. HIV status was determined through rapid testing (see above) and HCV status was self-reported. Indicators for social and physical vulnerability included homelessness, defined as currently homeless; trouble meeting basic needs (derived from research by Gelbert et al. [44]), defined as trouble finding a place to sleep, wash, use the bathroom or trouble having enough clothes or food to eat in the past 6 months; and income, categorized as income of <$900 or ≥$900 in the past month. Derived from the National Institute on Drug Abuse’s validated Risk Behavior Assessment [45], risky injection or sexual behavior was defined as receptive syringe sharing or unprotected sex with more than 1 partner in the past 6 months.

Our primary exposure variable for criminal justice involvement included two variables: (a) incarceration, defined as spending time in city jail, county jail or federal prison since the age of 18, and (b) community supervision, defined as having ever been on probation or parole. The primary exposure variable for substance use treatment involvement was defined as having ever received methadone detoxification, methadone maintenance, buprenorphine or suboxone, residential treatment (containing counseling, group therapy or cognitive behavioral therapy) or other outpatient treatment (containing counseling, group therapy or cognitive behavioral therapy).

Derived from the Urban Health Study questionnaire [46–49]—a community-based study with people who use drugs in Oakland for 15 years—other covariates of interest as potential confounders included current age; sexual risk group: categorized as men who have sex with women, men who have sex with men or men and women, women non-sex workers, women sex workers and transgender people; race/ethnicity: defined as African American, Caucasian, Latino/a, or Mixed Race/Other; high school education: defined as having received a high school diploma or GED; steady partnership: current relationship with a steady partner; having children and a history of injection drug use. The measures for social and physical vulnerability, criminal justice involvement, substance use treatment involvement were included as explanatory variables in assessing associations with HCV and HIV status.

### Statistical analysis

Descriptive statistics, including frequencies, median and interquartile range, were calculated to describe the distribution of variables in the study population. We calculated the prevalence and accompanying 95% confidence intervals for HIV and HCV status. Poisson regression models with robust variances were built to estimate adjusted prevalence ratios [42]. Our primary analysis of interest included assessing the impact of criminal justice and substance use treatment involvement with metrics for HCV testing and the steps of the HIV treatment cascade, risky behavior and social and physical vulnerability. Socio-demographic covariates considered for the multivariable model were determined based on their theoretical ability to confound the relationship of our exposures and outcomes. Backward stepwise regression with a criterion p value of 0.2 identified potential covariates for inclusion in the final multivariable model. Additionally, we examined the associations of socio-demographic, criminal justice and substance use treatment involvement and vulnerability with HIV and HCV status. Variables having a p value <0.2 with HIV or HCV status in bivariate analyses were considered for inclusion in the multivariable models, using backward stepwise regression with a criterion p value of 0.2. Statistical significance was set at p = 0.05. All statistical analyses were conducted in Stata v14 [50].

## Results

### Study population

A total of 2072 people who smoked crack cocaine or injected drugs were included in this analysis. Table 1 outlines characteristics of our study population. The median age of respondents was 49 years [interquartile range (IQR) 41–55], and nearly 60% were male. Regarding our exposures of interest, 92, 86 and 66% had been incarcerated since the age of 18, ever been in community supervision and ever involved in substance use treatment, respectively.

**Table 1:** Characteristics of people who inject drugs or smoke crack cocaine, 2011–2013 (N = 2072)

	HIV-negative (n = 2004)	HIV-positive (n = 68)	Total (n = 2072)
Age			
18–29	139 (7)	0 (0)	139 (7)
30–39	274 (14)	7 (10)	281 (13)
40–49	644 (32)	28 (41)	672 (32)
50–59	726 (36)	27 (40)	753 (36)
≥60	222 (11)	6 (9)	228 (11)
Male	1177 (59)	39 (57)	1216 (59)
Men who have sex with men	46 (2)	16 (23)	62 (3)
Men who have sex with women	1129 (56)	23 (34)	1152 (56)
Female	827 (41)	25 (37)	852 (41)
Non-sex worker	485 (24)	21 (31)	506 (24)
Sex worker	342 (17)	4 (6)	346 (17)
Transgender	1 (<1)	4 (6)	5 (<1)
Racial/ethnic group			
African American	1721 (86)	63 (93)	1784 (86)
Caucasian	100 (5)	3 (4)	103 (5)
Latino	76 (4)	0 (0)	76 (4)
Black Latino	10 (<1)	0 (0)	10 (<1)
Asian/Pacific Islander	8 (<1)	0 (0)	8 (<1)
Native American	13 (<1)	0 (0)	13 (<1)
Mixed	47 (2)	2 (3)	49 (2)
Other	30 (1)	0 (0)	30 (1)
High school education	1260 (63)	43 (63)	1303 (63)
Steady partnership	967 (48)	36 (53)	1003 (48)
Have children	1597 (80)	50 (73)	1647 (79)
Drug use history			
Injected drugs (only)	42 (2)	1 (<1)	43 (2)
Injected opioids (only)	31 (1)	0 (0)	31 (1)
Injected stimulants (only)	4 (<1)	1 (<1)	5 (<1)
Injected opioids and stimulants	7 (<1)	0 (0)	7 (<1)
Smoked crack/cocaine (only)	1476 (74)	50 (73)	1526 (74)
Injected drugs and smoked crack/cocaine	486 (24)	17 (<1)	510 (25)
Injected opioids and smoked crack/cocaine	245 (12)	5 (<1)	254 (12)
Injected stimulants and smoked crack/cocaine	26 (1)	6 (<1)	33 (2)
Injected opioids and stimulants and smoked crack/cocaine	215 (11)	6 (<1)	223 (11)
Homeless	1011 (50)	36 (53)	1047 (50)
Trouble meeting needs	1420 (71)	55 (81)	1475 (71)
Income <$900	1513 (76)	55 (82)	1568 (76)
Risky injection or sexual behavior	785 (40)	19 (28)	804 (39)
Incarcerated	1844 (92)	62 (91)	1906 (92)
Community supervision	1613 (86)	57 (89)	1670 (86)
Substance use treatment	1330 (67)	46 (68)	1376 (66)

**Fig. 1:**
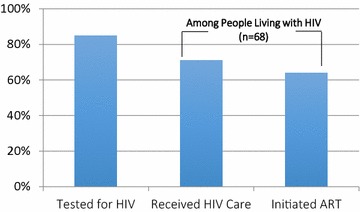
The continuum of HIV care for people who inject drugs or smoke crack cocaine

In terms of engagement in HIV care, 85% had ever been tested for HIV prior to study participation, and the HIV prevalence was 3.3% (95% confidence interval (CI) 2.6–4.1). Among people living with HIV (n = 68), 71% reported having ever received HIV care, and 64% reported having ever initiated antiretroviral therapy (ART) (Fig. 1). With regards to HCV testing, 65% had ever been tested for HCV, and the self-reported HCV prevalence was 31% (95% CI 29–34%).

### Criminal justice involvement

Criminal justice involvement was statistically associated with several HIV/HCV service access, vulnerability and risk behavior variables. Of note, people who reported a history of incarceration since the age of 18 were more likely to report testing for HIV ever (adjusted prevalence ratio (aPR) = 1.21; 95% CI 1.10–1.34; p < 0.001) and testing for HCV ever (aPR = 1.32; 95% CI 1.12–1.56; p = 0.001). Among people living with HIV, no statistically significant associations were observed between reported history of incarceration and receipt of HIV care or initiation of ART (Table 2). In addition, people who reported a history of incarceration were significantly more likely to report homelessness (1.22; 95% CI 1.00–1.48; p = 0.049), having trouble meeting basic needs (aPR = 1.14; 95% CI 1.01–1.28; p = 0.031) and risky injection or sexual behavior (aPR = 1.31; 95% CI 1.04–1.66; p = 0.024) (Table 3).

**Table 2:** Associations of criminal justice involvement and substance use treatment on HCV testing and steps of engagement in HIV care

	HCV testing		HIV testing		HIV care		Initiated ART	
	aPR (95% CI)	p value	aPR (95% CI)	p value	aPR (95% CI)	p value	aPR (95% CI)	p value
Incarceration	1.32 (1.12–1.56)	p = 0.001	1.21 (1.10–1.34)	p < 0.001	0.82 (0.52–1.29)	p = 0.394	0.79 (0.48–1.28)	p = 0.336
Community supervision	1.15 (1.04–1.29)	p = 0.009	1.17 (1.09–1.25)	p = 0.001	1.35 (0.59–3.07)	p = 0.470	2.04 (0.64–6.49)	p = 0.227
Substance use treatment	1.23 (1.14–1.33)	p < 0.001	1.15 (1.10–1.20)	p < 0.001	1.67 (1.10–2.55)	p = 0.016	1.92 (1.15–3.21)	p = 0.012

Age, gender, race, education, steady partnership, having children and injection drug use history assessed as potential confounders in all analyses

**Table 3:** Associations of criminal justice involvement and substance use treatment on vulnerability and risk behavior

	Homelessness		Trouble meeting needs		Income ≥ $900		Risky injection or sexual behavior	
	aPR (95% CI)	p value	aPR (95% CI)	p value	aPR (95% CI)	p value	aPR (95% CI)	p value
Incarceration	1.22 (1.00–1.48)	0.049	1.14 (1.01–1.28)	0.031	1.14 (0.84–1.55)	0.391	1.31 (1.04–1.66)	0.024
Community supervision	1.33 (1.14–1.55)	<0.001	1.12 (1.02–1.23)	0.014	1.22 (0.96–1.56)	0.104	1.18 (1.00–1.40)	0.048
Substance use treatment	1.05 (0.96–1.15)	0.245	1.00 (0.94–1.06)	0.952	1.19 (1.00–1.41)	0.045	1.10 (0.98–1.23)	0.119

Age, gender, race, education, steady partnership, having children and injection drug use history assessed as potential confounders in all analyses

Like people who have a history of incarceration, people who reported a history of community supervision were more likely to report testing for HIV ever (aPR = 1.17; 95% CI 1.09–1.25; p = 0.001) and testing for HCV ever (aPR = 1.15; 95% CI 1.04–1.29; p = 0.009). No statistically significant associations were observed between reported history of community supervision and receipt of HIV care or initiation of ART (Table 2). In addition, people who reported a history of community supervision were significantly more likely to report homelessness (aPR = 1.33; 95% CI 1.14–1.55; p < 0.001), having trouble meeting basic needs (aPR = 1.12; 95% CI 1.02–1.23; p = 0.014) and risky injection or sexual behavior (aPR = 1.18; 95% CI 1.00–1.40; p = 0.048) (Table 3).

### Substance use treatment

With regards to SUT, people who reported a history of substance use treatment were more likely to report receiving HIV testing ever (aPR = 1.15; 95% CI 1.10–1.20; p < 0.001) and HCV testing ever (aPR = 1.23; 95% CI 1.14–1.33; p < 0.001). Among people living with HIV, those who reported a history of SUT were more likely to report receiving HIV care (aPR = 1.67; 95% CI 1.10–1.25; p = 0.016) and initiating HIV treatment (aPR = 1.92; 95% CI 1.15–3.21; p = 0.012), as compared to people who had no history of SUT (Table 2). People who reported a history of SUT were more likely to report a current income ≥$900 (aPR = 1.19; 95% CI 1.00–1.41; p = 0.045); however, no statistically significant associations were observed between reported history of SUT and homelessness, trouble meeting needs or risky behavior (Table 3).

### Predictors of HIV and HCV status

In multivariable analysis of HIV seropositive status, men who reported having sex with men (aPR = 12.49; 95% CI 6.95–22.44; p < 0.001) and people who reported being transgender (aPR = 38.32; 95% CI 20.10–73.03; p < 0.001) had a higher likelihood of being HIV-positive. With regards to HCV, people who reported being older in age (aPR = 1.47 per 10 years; 95% CI 1.35–1.59; p < 0.001), men who have sex with men (aPR = 1.74; 95% CI 1.32–2.31; p < 0.001), transgender (aPR = 1.70; 95% CI 1.12–2.57; p = 0.010), Caucasian (aPR = 1.41; 95% CI 1.16–1.72); p < 0.001), drug injectors (aPR = 7.06; 95% CI 5.17–9.64; p < 0.001) and homeless [aPR = 1.30; 95% CI 1.14–1.48; p = 0.001) had a higher likelihood of self-reporting being HCV-positive (Table 4).

**Table 4:** Associations with HIV and HCV positivity among PWID and PWSC in Oakland, 2011–2013 (N = 2072)

	HIV-positive				HCV-positive			
	PR (95% CI)	p value	aPR (95% CI)	p value	PR (95% CI)	p value	aPR (95% CI)	p value
Age (per 10 years)	1.11 (0.99–1.03)	0.208			1.65 (1.52–1.80)	<0.001	1.47 (1.35–1.59)	<0.001
Sexual risk group								
Men who have sex with women	1.00 (ref)		1.00 (ref)		1.00 (ref)		1.00 (ref)	
Men who have sex with men	12.93 (7.20–23.20)	<0.001	12.49 (6.95–22.44)	<0.001	1.57 (1.15–2.14)	0.002	1.74 (1.32–2.31)	<0.001
Female non-sex workers	2.08 (1.16–3.72)	0.014	1.71 (0.98–2.99)	0.058	0.84 (0.68–1.04)	0.107	1.06 (0.89–1.25)	0.513
Female sex worker	0.58 (0.20–1.66)	0.310	0.56 (0.19–1.60)	0.277	0.86 (0.68–1.08)	0.197	1.21 (0.98–1.48)	0.069
Transgender	40.07 (22.07–72.76)	<0.001	38.32 (20.10–73.03)	<0.001	0.99 (0.20–4.96)	0.997	1.70 (1.12–2.57)	0.010
Racial/ethnic group								
African American	1.00 (ref)				1.00 (ref)		1.00 (ref)	
Caucasian	0.83 (0.26–2.60)	0.748			2.06 (1.67–2.54)	<0.001	1.41 (1.16–1.72)	<0.001
Latino	NC				1.88 (1.46–2.42)	<0.001	1.25 (0.99–1.59)	0.060
Mixed race/other	0.57 (0.14–2.29)	0.429			1.22 (0.88–1.70)	0.232	1.00 (0.73–1.37)	0.997
High school education	1.01 (0.62–1.65)	0.810			0.94 (0.80–1.10)	0.452		
Steady partnership	1.20 (0.75–1.91)	0.448			0.81 (0.69–0.96)	0.012		
Have children	0.72 (0.42–1.22)	0.217			1.01 (0.83–1.24)	0.896		
Ever injected drugs	1.19 (0.74–1.90)	0.470			8.98 (6.63–12.16)	<0.001	7.06 (5.17–9.64)	<0.001
Homeless	1.10 (0.69–1.76)	0.686			1.21 (1.03–1.42)	0.017	1.30 (1.14–1.48)	<0.001
Trouble meeting needs	1.71 (0.94–3.12)	0.077	1.61 (0.91–2.85)	0.101	1.27 (1.05–1.54)	0.012		
Income ≥ $900	0.69 (0.37–1.27)	0.230			1.16 (0.98–1.38)	0.088		
Incarcerated	0.87 (0.38–1.99)	0.746			2.45 (1.37–4.39)	0.002		
Community supervision	1.34 (0.62–2.90)	0.464			1.41 (1.05–1.88)	0.022		
Substance use treatment	1.06 (0.64–1.74)	0.826			1.91 (1.53–2.39)	<0.001	1.20 (0.99–1.45)	0.060

NC: model did not converge for this category

## Discussion

With data from 2072 PWSC and PWID, people who reported a history of SUT were more likely to report higher levels of HIV and HCV screening, HIV care and ART initiation and were not more likely to report increased vulnerability or risky behavior. Other research has also suggested that people with a history of SUT have improved access to HIV care and treatment [30–33]. Researchers have also suggested that SUT can attend to the root causes of drug-related offenses among drug-involved offenders [28]. Yet as of 2011, 21.6 million people aged 12 or older had a substance use disorder, but only 2.3 (11%) million people received SUT [51]. There remains a great opportunity to invest and expand the use of SUT for drug-involved people with criminal justice histories in order to attend to their substance use disorder [28] and facilitate access to other essential health care such as HIV care and treatment.

In addition, our results suggested that people with a history of criminal justice involvement reported greater access to HIV and HCV testing, but did not report greater access to HIV care or treatment. Researchers and practitioners have urged medical and public health professionals to take advantage of the opportunities provided by the criminal justice system in providing care for a highly underserved population [16, 52, 53]. Both the California state prison and Alameda county jail systems have an opt-out model for HIV testing, and the community supervision system provides testing referrals as indicated by the court or probation/parole officer. While our study did not evaluate these specific policies, it is encouraging that a history of criminal justice involvement was associated with higher reported levels of testing.

Accessing HIV services can quickly become complicated and disjointed for people with a history of criminal justice involvement. People incarcerated for longer sentences might be able to reap the benefits of HIV care and treatment during incarceration, but lose them once released [54]. Further, people who are going in and out of jail for low-level drug use or ‘quality of life’ violations are likely to miss any stabilizing benefit as they are caught in a criminal justice cycle that continually changes their environment. Our findings suggest that people with a history of criminal justice system involvement do not have greater access to HIV care or treatment.

Our findings are also consistent with previous research suggesting that involvement with the criminal justice system is associated with increased social and physical vulnerability [55, 56]. Prior studies have found an increased likelihood of homelessness [57], unemployment [58], lack of educational opportunities [55] and lack of health insurance [59] among people who have been involved in the criminal justice system. Furthermore, our findings showed increased injection- and sexual-related risk behavior among people with a history of criminal justice involvement is consistent with previous research that suggests this dynamic can be driven by factors such as poverty, mental health disorders and concurrent sexual partnerships, that are a by-product of involvement with the criminal justice system [55, 60].

The principal limitation of the study is the observational nature of the research design. Although we adjusted for measured participant characteristics to address concerns of confounding, the potential for unmeasured or mismeasured factors to bias our results existed. In addition, concerns regarding the temporality between exposures and outcomes given the cross-sectional nature of our study exist. We attempted to ameliorate this concern by defining exposure periods prior to outcomes when possible, but this does not convey the same level of rigor as a longitudinal study. Our study had the largest sample and most power for the earlier stage of the HIV care continuum (testing), and included a small number of people living with HIV, which may have impacted the study’s power to detect associations for the following stages of the continuum (receiving HIV care and initiating ART). Furthermore, this study was not designed to look for relationships between different types of substance use treatment for different substance use disorders on study outcomes.

Another limitation is the self-report of behaviors and HCV status for the survey, and as a result, recall and social desirability are potential biases impacting the metrics collected as part for this analysis. Specifically, metrics for vulnerability (e.g., homelessness), sexual risk factors (e.g., multiple partners), injection risk factors (e.g., sharing needles) may be prone to social desirability bias. Due to stigmatization of behaviors and conditions, these types of biases are common when studying people who use drugs [61]. However, the resulting misclassification would be non-differential with regards to our primary exposures of interest and would generally bias our results toward the null [62]. On the other hand, prior research has illustrated that self-reported behaviors from drug users are both reliable and valid for epidemiological purposes [63].

Strengths of the study included the community-recruited sample of study participants. Most studies of HIV care engagement typically use service utilization data, while our sample provides a snapshot of access to services among people recruited from a community setting. Interviewing participants in community settings that are independent of any criminal justice or substance abuse treatment institution may increase participants’ comfort with disclosing stigmatizing behavior compared to participants who are interviewed within institutional settings. In addition, our study included a large sample of criminal justice involved individuals who would not be captured in prison or jail populations, including people who were on probation or parole, who had absconded, or who had warrants out for their arrest. Furthermore, we had data of high quality that were collected specifically for the purposes of research.

## Conclusion

Challenges remain in improving access to components of the HIV continuum and reducing vulnerabilities and risk among PWSC and PWID who have a history of the criminal justice system involvement. Innovative models that improve access to the HIV care continuum for these populations is vital. In addition, utilizing SUT programs, as appropriate, that are integrated with other services for HIV, HCV and mental health disorders will be critical to simultaneously address poor access to HIV and HCV services and the underlying reasons people who use drugs engage in drug-related offenses.
